# Succinate dehydrogenase/complex II activity obligatorily links mitochondrial reserve respiratory capacity to cell survival in cardiac myocytes

**DOI:** 10.1038/cddis.2015.310

**Published:** 2015-10-29

**Authors:** R Dhingra, L A Kirshenbaum

**Affiliations:** 1 Institute of Cardiovascular Sciences, St Boniface Hospital Research Centre, University of Manitoba, Winnipeg, Manitoba, Canada; 2 Department of Physiology and Pathophysiology, College of Medicine, Faculty of Health Sciences, University of Manitoba, Winnipeg, Manitoba, Canada

The concept that cells die by a genetically regulated process has profound implications for human diseases in which too little or too much cell death is the primary etiology of the disease. In the context of the adult myocardium, which exhibits a limited capacity for *de novo* myocyte regeneration after injury, the functional loss of cardiac cells by de-regulated programmed apoptosis or necrosis is postulated as the central cause of ventricular remodeling and heart failure following myocardial infarction.^1, 2^

Although the mitochondrion has been identified as a central organelle for integrating substrate utilization and ATP synthesis for normal cellular function, it has also been identified as a critical signaling platform for initiating and executing cell death.^3, 4^ Indeed, while the relationship between cell metabolism, substrate utilization and death is profound, the metabolic sensors and regulators that couple mitochondrial substrate utilization for ATP synthesis and cell survival in cardiac cells remain cryptic. In contrast to other cells, cardiac myocytes rely on mitochondrial fatty acid oxidation as the principle fuel source for ATP synthesis. The issue of mitochondrial bioenergetics is of particular importance in the heart as there is a continual demand for high ATP levels by cardiac myocytes for generating contractile force. This is best exemplified by the increased ATP demand by cardiac myocytes during exercise, which require additional cellular energy to sustain the increased cardiac workload. Under normal basal conditions mitochondria produce sufficient ATP to fully meet the energy demands of the cell, but retain the capacity to increase ATP synthesis upon increased energy demand. This ability of mitochondria to increase ATP production commensurate with demand is referred to as ‘spare or reserve' respiratory capacity (RRC). The inability of the cell to sufficiently recruit RRC results in an energy deficit that culminates in cell death.

Despite the vast literature on adaptive mitochondrial programming linking AMP-activated protein kinase (AMPK) and pyruvate dehydrogenase (PDH) to mitochondrial oxidative metabolism in cardiac myocytes, there is a paucity of available information regarding how these metabolic sensors and regulators actually impact mitochondrial oxygen consumption rates (OCR), oxygen-linked ATP production and RRC. Moreover, even less is known of how substrate-metabolic associations link mitochondrial metabolism and respiratory capacity to cell survival during hypoxia or ischemic stress. Under normal aerobic conditions the heart generates ATP by mitochondrial oxidation of fatty acids as the principle fuel source, however, during hypoxia cardiac metabolism shifts from fatty acid to glucose metabolism where glycolysis is the principle source of ATP. Complete glucose oxidation involves the conversion of glucose via glycolysis to pyruvate, which is then fed into to the mitochondrial Krebs cycle (tricarboxylic acid, TCA) for generating electron donors NADH and FADH_2_ for driving electron transport chain (ETC) flux and mitochondrial ATP synthesis. The rate limiting step for this process is invariably the conversion of pyruvate to acetyl-CoA by mitochondrial PDH. Indeed, PDH is the gate-keeping step that regulates conversion of pyruvate to acetyl-CoA. In the absence of this critical conversion step pyruvate is metabolized in the cytoplasm to lactate. Notably, PDH is inhibited by pyruvate dehydrogenase kinase (PDK). Hence, elevated levels of PDK inhibit mitochondrial pyruvate flux and mitochondrial TCA-linked respiration by phosphorylating and inhibiting PDH. Though four isoforms of PDK have been identified, PDK2 and PDK4 isoforms are expressed in heart. How PDKs regulate PDH mitochondrial coupled oxidative metabolism and RRC in the heart remain undefined.

Herein, the manuscript by Pfleger *et al.* address this point in a series of well-designed and carefully executed studies. The authors demonstrate a connection between substrate utilization and mitochondrial respiratory activity. In fact, the seminal finding of the study is the demonstration that cellular substrate can directly influence RRC in a cell- and context-specific manner. For example, neonatal myocytes required both glucose and fatty acids for developing RRC, while human-induced pluripotent stem cell-derived myocytes required only fatty acids for developing RRC that was dampened by addition of glucose. This finding is concordant with metabolic phenotype of the adult heart. This metabolic dependence of RRC was further validated by the demonstration that the metabolic sensor AMPK and metabolic regulators PDH, and the NADH-dependent class III deacetylase Sirtuin-3, positively regulated RRC in cardiac myocytes. This finding is concordant with recent reports demonstrating the ability of RRC to be regulated by these metabolic sensors.^5, 6^ The data are novel because it demonstrates that RRC can be regulated in substrate-dependent manner in cardiac myocytes. Concordantly, the authors found that hypoxia compromised oxidation of substrates and abolished RRC, with only having incremental effects on basal OCR. Notably, activation of AMPK by AICAR or PDH via the inhibition of PDK4 with dichloroacetic acid (DCA), rescued RRC and OCR.

Despite these interesting findings, the underlying source of the mitochondrial RRC in cardiac myocytes remained elusive. In an attempt to address this point, the authors reasoned that RRC may be coupled to a ‘factor' that increases substrate flux coincident with increased metabolic demand on the cell through TCA and ETC flux. In a variety of complementary studies, the authors systematically tested this notion and identified the ‘x-factor' to be succinate dehydrogenase (Sdh). Notably, Sdh is synonymous with complex II of the ETC. Indeed, the exciting feature of this finding is that Sdh is the only enzyme commonly shared between the TCA cycle and ETC thereby providing the ‘missing link' that couples substrate utilization to mitochondrial ETC and respiration. Perhaps most compelling evidence to support Sdh as the elusive factor ‘x' for regulating RRC was the demonstration that RRC was completely abolished by inhibiting the Sdh catalytic activity or by genetic knockdown of the Sdh assembly factor Sdhaf1, which is required for complex II activity. Hence, these findings not only substantiate the importance of complex II in TCA cycle but also importantly provide a functional link between complex II and RRC,^7^ Figure 1. Although it is generally believed that an increased energy demand will utilize RRC to increase ATP synthesis, this point had not been proven. The authors show that, indeed, this effect is accentuated by the activation of AMPK in cardiac myocytes, in which the RRC is displaced with almost a twofold increase in basal ATP synthesis-linked OCR under these conditions. Importantly, the observed increase was dependent on complex II activity, which corresponded directly with cell survival. The data provide a novel mechanism to explain on how cellular bioenergetics, in particular RRC, functionally increase ATP synthesis after hypoxic stress. Ostensibly, the increased RRC provides a survival mechanism by replenishing ATP lost during hypoxic stress. Hence, based on the present study, one can infer that identifying the optimal substrates for a given cell type, RRC, could be manipulated as a mechanism for enhancing ATP and cell survival during stress. In this regard, it would be interesting to test whether altered metabolic substrates would restore impaired RRC in human fibroblasts of valosin-containing protein-associated multisystem proteinopathy patients,^8^ in patients with perioperative lymphopenia ^9^ or influence the adaptability of human adipocytes to hypoglycemia.^10^

Collectively, the current data together with earlier work in neuron and fibroblasts demonstrate impaired RRC correlates with increased cell death^11, 12, 13^ and support the notion that loss of RRC may underlie disease. Thus, optimizing metabolic substrate availability, by enhancing glucose or fatty acid oxidation by either inhibiting PDKs or activating AMPK, respectively, may prove beneficial in increasing RRC and survival. Alternatively, increasing complex II stability during cellular stress may provide a more direct approach for enhancing or preserving RRC.

Although the authors have demonstrated a role for complex II in RRC, the possibility exists that defects in RRC during cellular stress such as hypoxia may directly or indirectly influence RRC. Alternatively, limited availability of TCA reducing intermediates entering the ETC could potentially impair RRC. For example, the depletion of one or more TCA substrate during cellular stress or high metabolic demand during exercise could compromise complex II activity and RRC. As complex II is comprised of multiple subunits, mutations in one or more of these subunits could presumably impair RRC and ATP synthesis. It would therefore be interesting to test whether complex II defects underlie the impaired RRC and ATP deficit commonly seen in human hearts with pre-existing mitochondrial disease. Another area of interest is the relationship between AMPK as a metabolic sensor of cellular stress and complex II activity, and remains unexplored. Notably, as mTOR that drives cellular processes associated with mitochondrial biogenesis and cell growth is regulated by AMPK, it would be important to test whether mitochondrial biogenesis during cardiac hypertrophy in response to systemic metabolic conditions such as diabetes influence RRC and cell survival. Interestingly, Sirt-3 knockdown inhibited AICAR- and DCA-induced RRC, it would be interesting to know whether Sirt-3 influences RRC by deacetylating one or more complex II proteins.

Further, given the observed differences in RRC and substrate utilization between neonatal cardiac cells and human iPS cells, it remains untested whether the observed effects on RRC by complex II is universally conserved or a restricted feature of cardiac myocytes. For example, it would be interesting to test whether cancer cells that preferentially generate ATP through aerobic glycolysis would exhibit similar regulation of RRC by complex II during nutrient or hypoxic stress.

Nevertheless, under the conditions tested the authors provide new compelling evidence that RRC and cell survival are regulated by mitochondrial complex II activity in a substrate and cell type-dependent manner. The study fundamentally may explain how mitochondrial metabolism linked through complex II activity influences RRC, ATP production and cell survival of cardiac myocytes during hypoxic stress.

## Figures and Tables

**Figure 1:**
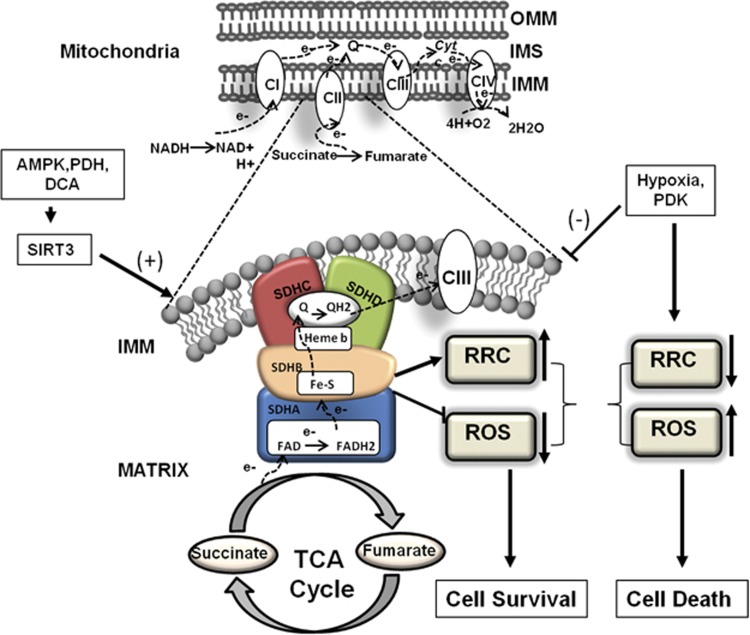
Mitochondrial respiratory complex II regulates reserve respiratory capacity and cell survival. Pfleger *et al.* provides evidence that complex II (Sdh) regulates RRC and cell survival of ventricular cardiomyocytes. Top, schematic representation of electron flow through mitochondrial ETC (CI, II, III and IV refer to mitochondrial ETC complexes I, II, III and IV; IMM, inner mitochondrial membrane; IMS, intermembrane space; NADH, ubiquinone oxidoreductase; OMM, outer mitochondrial membrane). Bottom, detailed structural view of mitochondrial respiration complex II, which is a component of both the TCA cycle and respiratory ETC; it couples substrate utilization to mitochondrial ETC and respiration. Complex II is comprised of four subunits: SDHA, SDHB, SDHC and SDHD, and is the source of RRC, which is critical for cell survival. In this editorial the authors demonstrate that complex II regulates RRC by increasing substrate flux coincident with increased metabolic demand on the cell through TCA and ETC flux. Hypoxia and PDK negatively regulate complex II activity resulting into diminished RRC, ROS and cell death. Activation of AMPK by AICAR or PDH via the inhibition of PDK4 with DCA, rescued RRC, OCR and cell death of cardiomyocytes during hypoxia. Importantly RRC induced by AICAR and DCA is mediated via the class III NAD-dependent deacetylase Sirtuin-3
